# Photodynamic reactions using high-intensity red LED promotes gingival wound healing by ROS induction

**DOI:** 10.1038/s41598-023-43966-2

**Published:** 2023-10-10

**Authors:** Emika Minagawa, Nobuhiro Yamauchi, Yoichiro Taguchi, Makoto Umeda

**Affiliations:** Department of Periodontology, Osaka Dental University, 8-1 Kuzuhahanazono-cho, Hirakata, Osaka Japan

**Keywords:** Cell growth, Periodontitis, Lasers, LEDs and light sources

## Abstract

Photodynamic therapy is a treatment that combines a light source with a photosensitizer. LEDs have attracted considerable attention in clinical dentistry because they are inexpensive and safe to use. Although the interaction between photosensitizers and LEDs in dental practice is effective for treating periodontal disease by killing periodontopathic bacteria, little is known about the effects of LEDs on human gingival fibroblasts (HGnFs), which play an important role in gingival wound healing. In this study, we investigated the effects of high-intensity red LED irradiation on HGnFs after the addition of methylene blue (MB), one of the least harmful photosensitizers, on wound healing and reactive oxygen species (ROS) production induced by photodynamic reactions. We found that irradiation of MB with high-intensity red LED at controlled energy levels promoted cell proliferation, migration, and production of wound healing factors. Furthermore, ROS production by a photodynamic reaction enabled the translocation of phosphorylated Grb2-associated binder-1, activating Extracellular signal-regulated kinase 1/2 and c-Jun N-terminal kinase signals. Our findings suggest that proper control of ROS production has a beneficial effect on gingival fibroblasts, which constitute periodontal tissue, from the perspective of gingival wound healing.

## Introduction

Periodontitis is caused by bacteria, their byproducts and the body's immune response. It is a multifactorial disease that destroys connective tissue and alveolar through the actions of numerous cytokines and enzymes^1,2^. The treatment of periodontitis is essential for reducing gingival inflammation by eliminating one of the causative periodontopathic bacteria, which promotes wound healing. Wound healing is a complex biological process that involves the repair of damaged tissues and aims to maintain the functional and anatomical continuity of the tissue^3^. It consists of three phases: an inflammatory phase, a proliferative phase, and a tissue remodeling phase^4^. This process involves cell proliferation, migration, and extracellular matrix (ECM) synthesis.

Human gingival fibroblasts (HGnFs) are one of the main constituents of gingival cells and play an important role in wound healing^5,6^. HGnFs promote healing by inducing angiogenesis and generating a new ECM. The densities of cells and ECM are crucial for wound healing and influence the state of the repaired tissue.

Light is used as a form of non-invasive therapy, and irradiation with red or near-infrared light of specific wavelengths produces various physiological effects on cells and tissues^7^. Light irradiation promotes cell proliferation^8^, wound healing^9^, and pain relief^10^. Although this type of therapy has been developed mainly using low-level lasers, LED have attracted attention as a new light source in recent years, owing to their cost-effectiveness, safety, and lack of the need for large-scale equipment. We previously reported that high-intensity red LED irradiation as a light source promotes bone differentiation potential in human periodontal ligament stem cells^11^ and human bone marrow stem cells^12^.

Photodynamic therapy (PDT) is effective for treating tissue and skin diseases by generating reactive oxygen species (ROS) through a photodynamic reaction that combines a light source and a photosensitizer. It is also utilized to kill periodontal pathogens in the treatment of periodontal disease^13^ and has garnered attention owing to the risk of bacterial resistance to antimicrobial agents and safety in patients with systemic disease. There are various types of photosensitizers, among which Methylene Blue (MB) is a water-soluble photosensitizer with a strong absorption band in the 550–700 nm (maximum absorbance peak: 665 nm) region, and is widely used because of its low toxicity and lack of side effects^14^. Previously, laser irradiation using MB as a photosensitizer was reported to have a bactericidal effect on the oral bacteria responsible for oral candidiasis^15^, periodontitis^16^, and peri-implantitis^17^. We have previously reported that irradiation with MB and a high-intensity red LED light source inhibits the growth of periodontopathic bacteria when appropriate irradiation energy is used^18^. However, PDT must consider its effects not only on the target bacteria but also on the surrounding tissues^14^. During the treatment of periodontal disease, photodynamic reactions are used to avoid damaging effects on periodontal tissues. However, considering the various beneficial effects of light, we explored the possibility of obtaining positive effects on the surrounding tissues by adjusting the irradiation energy of high-intensity red LED, instead of adverse or no effects.

ROS are produced by various stimuli and their excessive production has been reported to cause oxidative stress and cellular damage^19^, whereas low levels of ROS activate cellular functions through redox signaling^20^. Furthermore, ROS production has been reported to play an important role in regulating the wound healing response, as well as the induction of cells to the wound site and angiogenesis during the repair process^21^.

The mitogen-activated protein kinase (MAPK) pathway is a key intracellular signaling pathway involved in wound healing^22^. Low levels of ROS production have been reported to activate this pathway. Moreover, the activity of this pathway may be triggered by ROS transferring phosphorylated receptor tyrosine kinases (RTKs) to the cytoplasm^23^. Although the mechanism by which photodynamic reactions using high-intensity red LED as a light source induce cellular responses is unknown, it is possible that ROS production transmits the activity of RTKs into the cytoplasm independent of ligands, such as growth factors, by activating the MAPK pathway. Grb2-associated binder-1 (Gab1) is activated by binding to RTKs and serves as a scaffold for signaling into the cytoplasm^24–26^. It is normally activated by growth factor stimulation, but may also be activated by photodynamic reactions, potentially triggering MAPK pathway activity.

In this study, we investigated the effects of high-intensity red LED irradiation of HGnFs after the addition of MB on wound healing as well as its involvement in ROS production induced by photodynamic reactions.

## Results

### Optimal energy level determination

**Figure 1:**
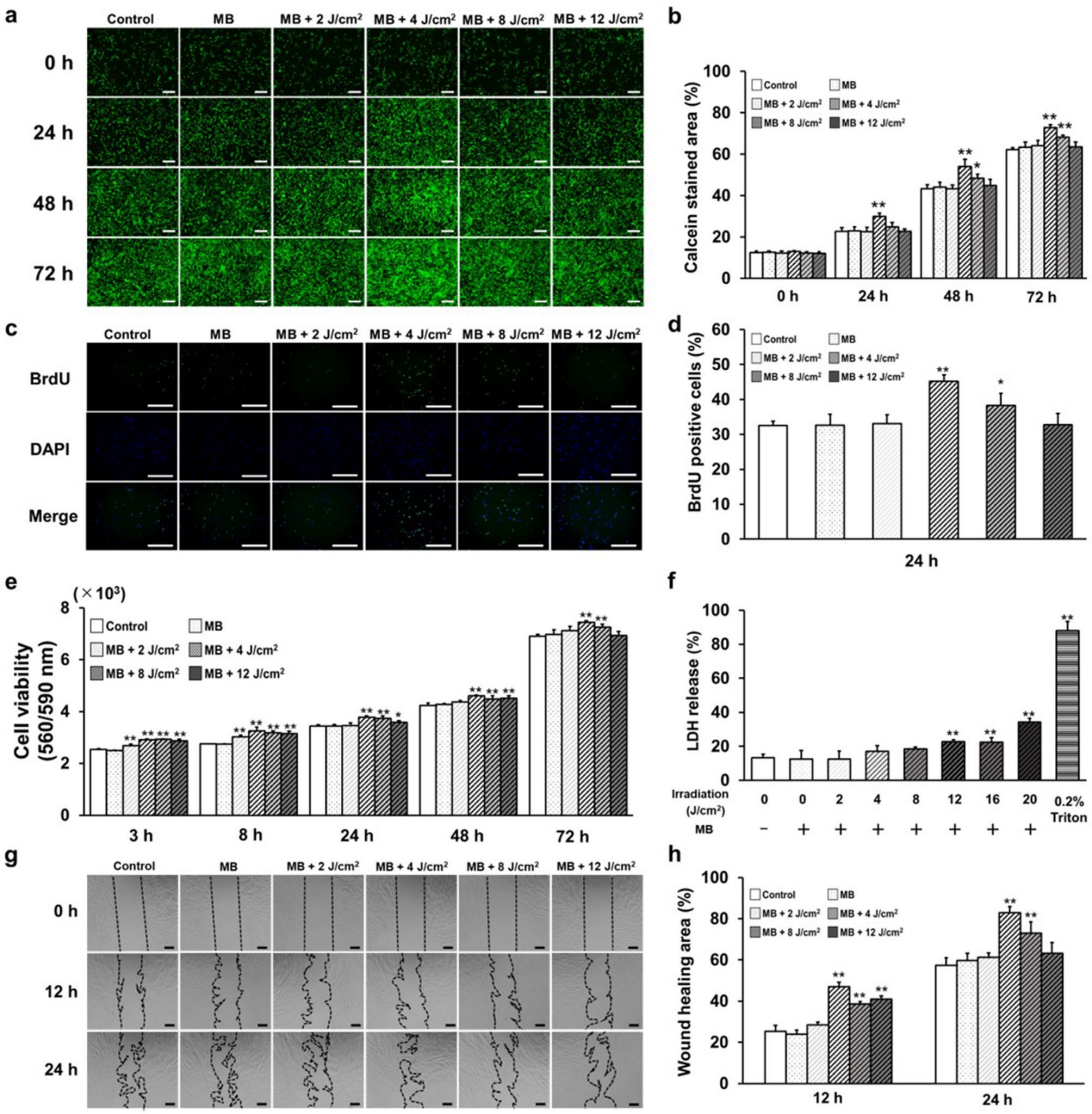
Effects of PDT irradiated at various energy doses on HGnFs. (**a**) Calcein staining was captured by fluorescence microscopy 0, 24, 48, and 72 h after stimulation. (**b**) Comparison of percentages of calcein-stained areas. (**c**, **d**) Representative immunofluorescence images of BrdU were incorporated into DNA, and the nuclei were stained by DAPI following a period of 24 h incubation after stimulation; data were compared by BrdU positivity rate. (**e**) Cell viability was measured after 3, 8, 24, 48, and 72 h of incubation. (**f**) LDH release was measured after 72 h. (**g**) Wound healing assays were performed at 0, 12, and 24 h. (**h**) Wound healing assay data represents the percentage of the cellular area at 12 and 24 h. (scale bar: 200 μm) Significant increases compared with the control: *p < 0.05, **p < 0.01.

The optimal energy levels were determined within an irradiation range of 0–12 J/cm^2^ after the addition of MB. After 0, 24, 48, and 72 h, viable cells were stained with calcein and the staining areas were compared. LED irradiation with MB induced the highest number of viable cells at 4 J/cm^2^ (Fig. 1a,b). Cell proliferation rate was highest at 4 J/cm^2^ after 24 h (Fig. 1c,d). Cell viability was the highest at 4 J/cm^2^ after 3, 8, 24, 48, and 72 h (Fig. 1e). In the cytotoxicity assay, the LDH release significantly increased above 12 J/cm^2^ (Fig. 1f). Cell migration at 12 and 24 h was evaluated by cell migration, and the resulting cell areas were compared, with the highest cell migration occurring at 4 J/cm^2^ (Fig. 1g,h). Therefore, a dose of 4 J/cm^2^ was used for the subsequent assays. MB alone showed no significant difference compared with the control.

### Wound healing assessment

**Figure 2:**
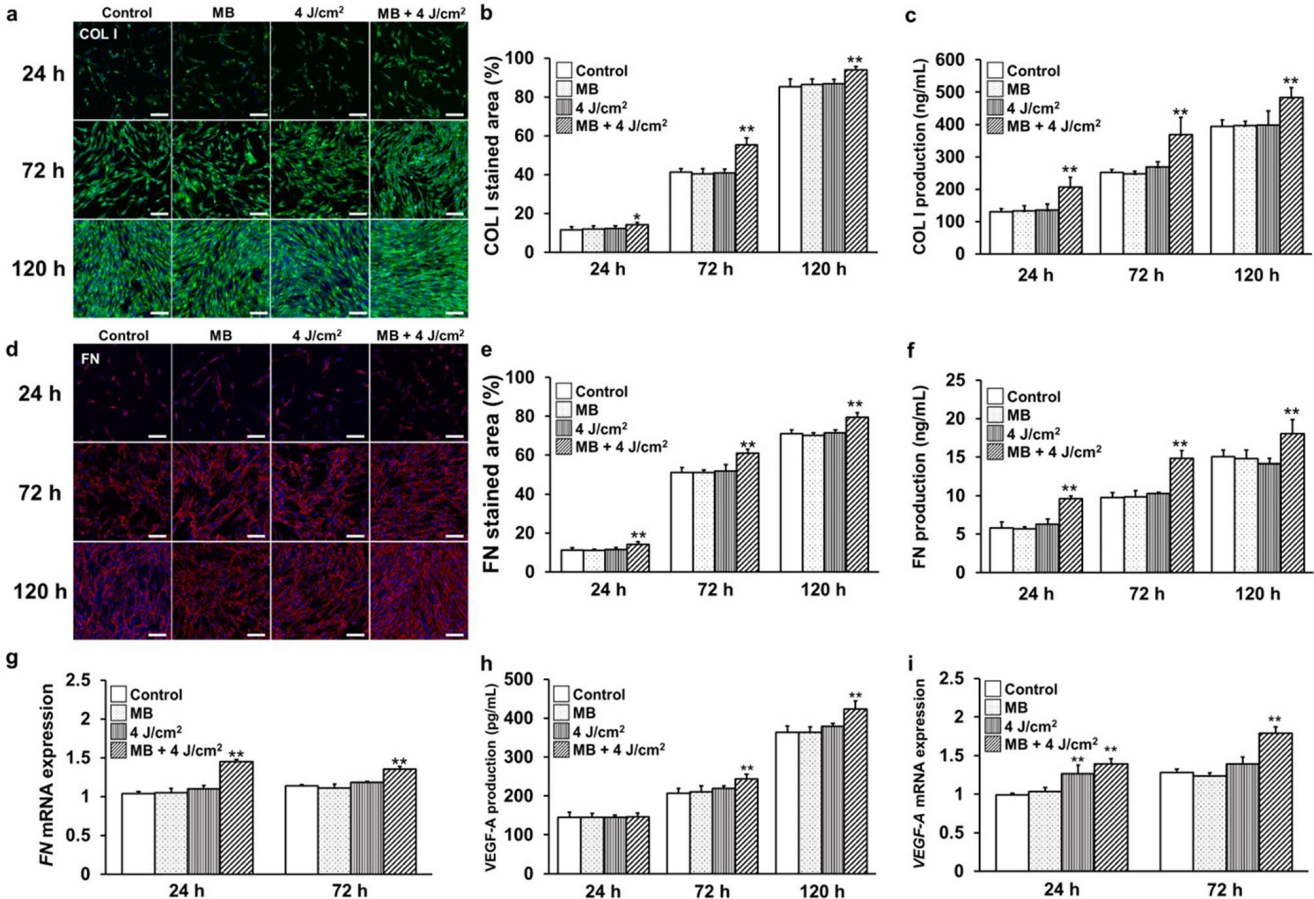
PDT enhances the wound-healing ability of gingival fibroblasts through the production of ECM. (**a**, **b**) Immunofluorescence staining of COL I was visualized by confocal laser microscopy after 24, 72, and 120 h of incubation; data for COL I are compared by the ratio of staining area. (**c**) COL I production was measured at 24, 72, and 120 h. (**d**, **e**) Immunofluorescence staining of FN was visualized by confocal laser microscopy after 24, 72 and 120 h of incubation; data of FN are compared by the ratio of staining area. (**f**) FN production was measured at 24, 72, and 120 h. (**g**) *FN* mRNA gene expression was measured at 24 and 72 h. (**h**) VEGF-A production was measured at 24, 72, and 120 h. (**i**) *VEGF-A* mRNA gene expression was measured at 24 and 72 h. (scale bar: 200 μm) Significant increases compared with the control: *p < 0.05, **p < 0.01.

Human type I collagen (COL I) (Fig. 2a) and fibronectin (FN) (Fig. 2d), the major components of the extracellular matrix (ECM) involved in wound healing, were evaluated using fluorescent immunostaining. The stained areas of the images were compared (Fig. 2b,e), and MB addition and LED irradiation showed significant increases after 24, 72, and 120 h. Additionally, COL I, FN, and the production of vascular endothelial growth factor-A (VEGF-A), an important factor that promotes angiogenesis in wound healing, were significantly increased in the supernatants after 24, 72, and 120 h (Fig. 2c,f,h). The expression of *FN* mRNA (Fig. 2g) and *VEGF-A* mRNA (Fig. 2i) also significantly increased after 24 h and 72 h.

### Intracellular signaling pathways

**Figure 3:**
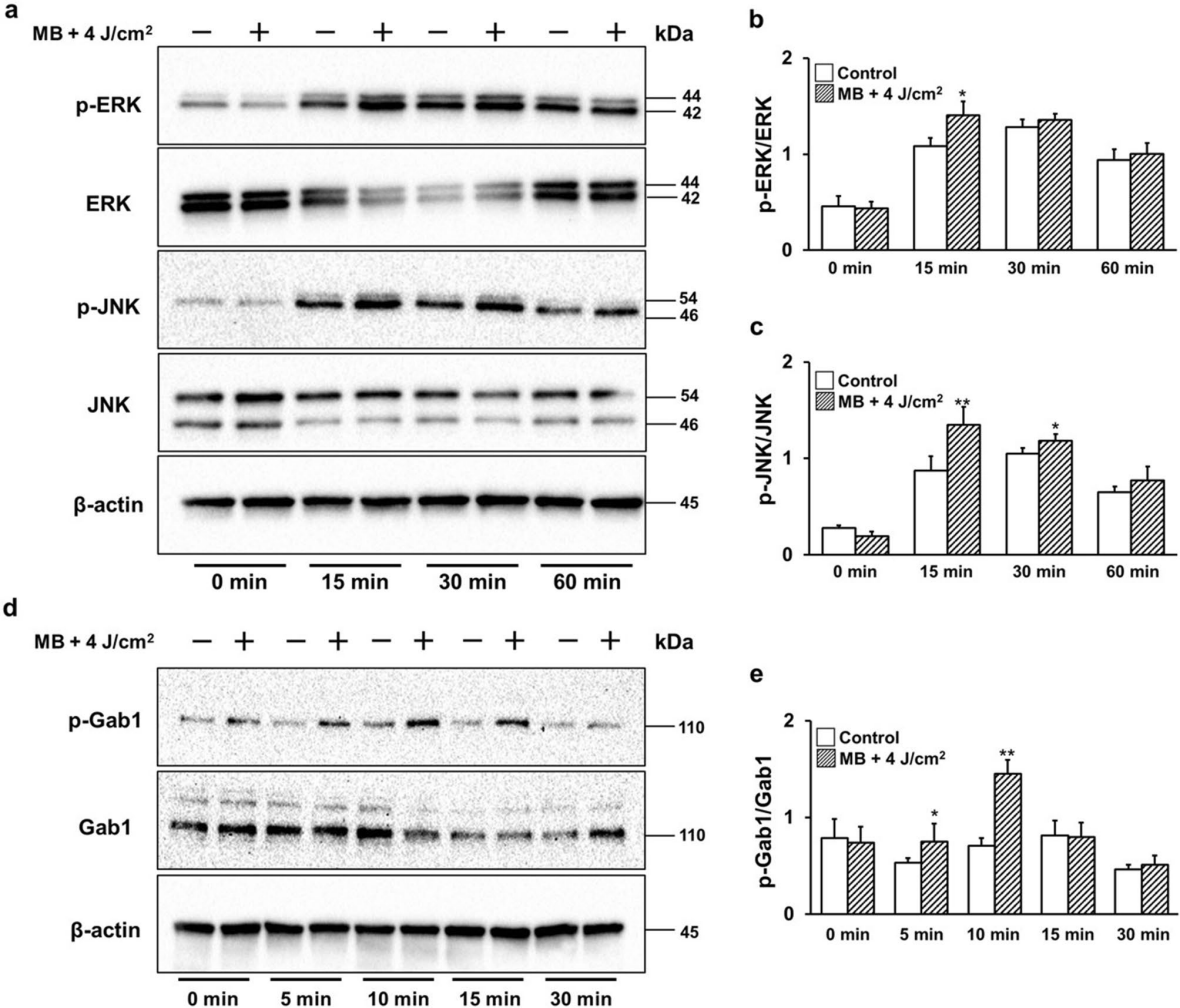
PDT on HGnFs activates the ERK1/2, JNK, and Gab1 signaling pathways. The expression levels of ERK1/2, JNK, and Gab1 were analyzed using western blotting (**a**). Western blotting was performed on the protein extracts of these cells with antibodies against the indicated proteins, using β-actin as a loading control. p-ERK and ERK expressions were quantified using ImageJ software (**b**). p-JNK and JNK expression levels were quantified using the ImageJ software (**c**). Western blot analysis (**d**) and quantification of p-Gab1 and Gab1 expression using ImageJ software (**e**). Western blot analysis was performed on protein extracts of these cells with antibodies against the indicated proteins with β-actin as a loading control. The samples derive from the same experiment and those gels/blots were processed in parallel. Uncropped blots for this experiment are presented in supplementary file 1. Significant increases compared with the control: *p < 0.05, **p < 0.01.

Next, activation of the MAPK pathway, an important intracellular signal in wound healing, was assessed. Extracellular signal-regulated kinase (ERK) 1/2 was significantly activated 15 min after MB addition and LED irradiation (Fig. 3a,b). The activation of c-Jun N-terminal kinase (JNK) was significantly upregulated 15 and 30 min after MB addition and LED irradiation (Fig. 3a,c). Gab1 transduces RTKs into cells and activates the downstream MAPK pathway. Gab1 was significantly activated 10 min after MB addition and LED irradiation (Fig. 3d,e).

### Involvement analysis of ROS induction

**Figure 4:**
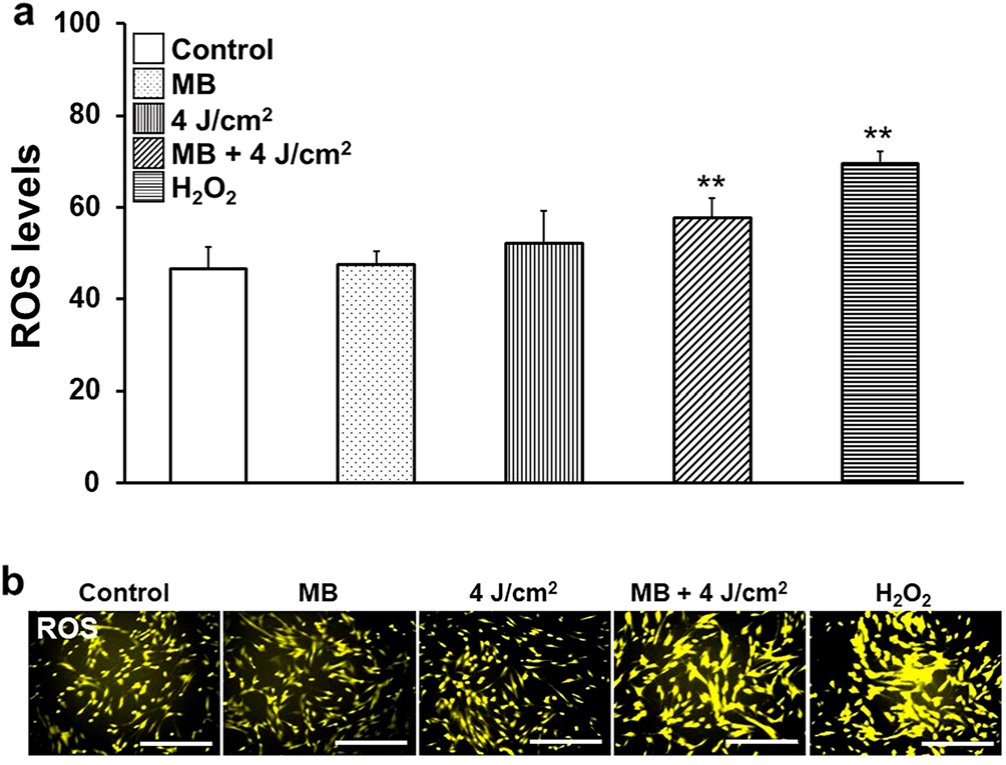
HGnFs produce ROS via photosensitization. ROS levels were examined after stimulation with a total ROS kit. (**a**) Fluorescence intensity was measured using a plate reader, and the data were compared to those of the control. HGnFs were stimulated with H_2_O_2_ (1 µM) as a positive control. (**b**) Fluorescent staining was performed using a fluorescence microscope. (scale bar: 200 μm). Significant increases compared with the control: *p < 0.05, **p < 0.01.

The ROS produced by photodynamic reactions with a high-intensity red LED were measured and stained. After stimulation, ROS was significantly detected following the addition of MB addition and LED irradiation (Fig. 4a,b).

**Figure 5:**
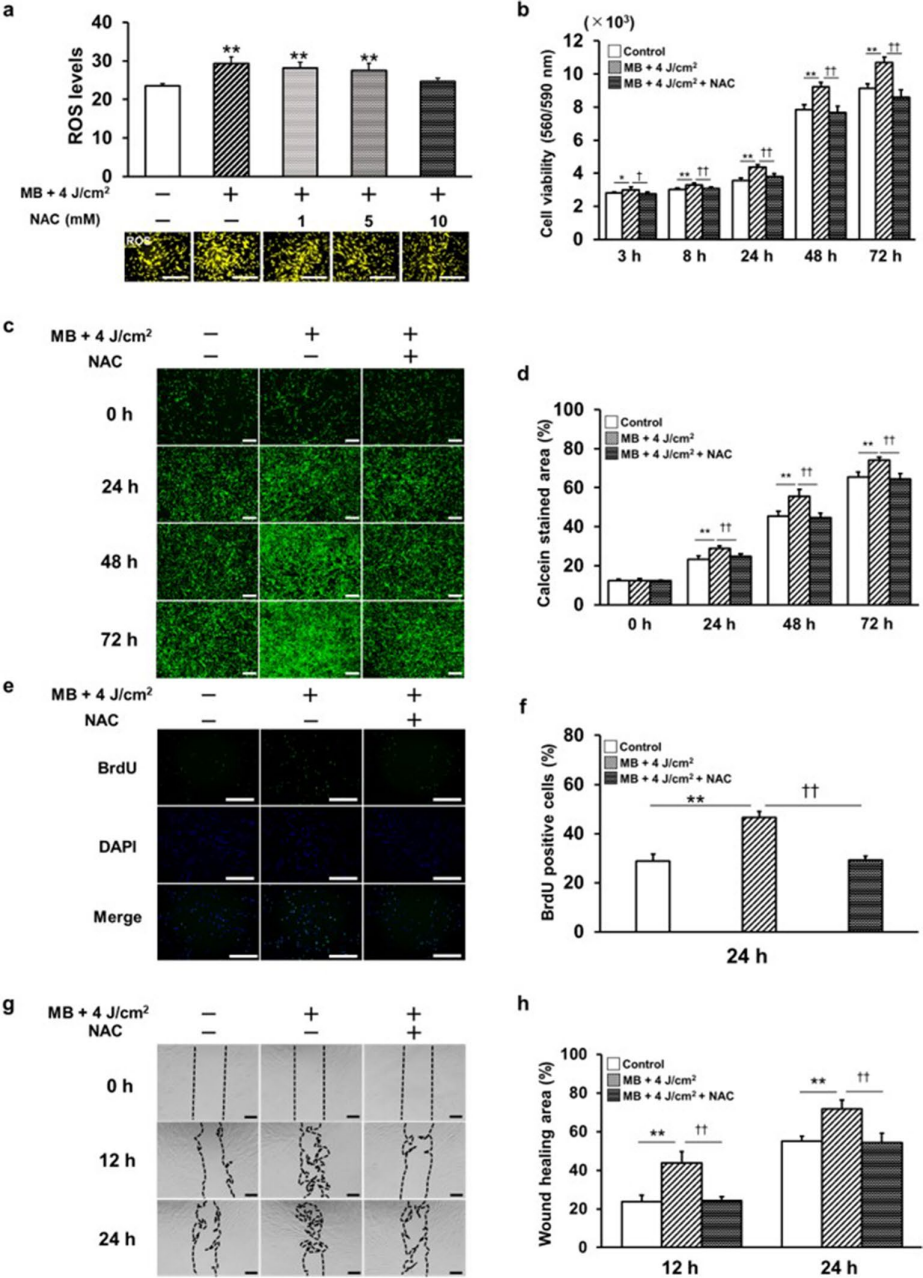
The cell-active capacity of HGnFs, such as cell proliferation and migration, is controlled by the production of ROS, which is influenced by NAC. NAC (1, 5, and 10 mM) was added to the MB medium, and ROS levels were examined after PDT. (**a**) Fluorescence staining was performed using a fluorescence microscope. Fluorescence intensity was measured using a microplate reader, and the data were compared with those of the control. (**b**) Cell viability was measured after 3, 8, 24, 48, and 72 h of incubation. (**c**) Calcein staining was visualized using fluorescence microscopy after 0, 24, 48, and 72 h of incubation. (**d**) The percentages of calcein-stained areas were compared. (e) Cell proliferation was assessed using fluorescence immunostaining after 24 h of incubation. (**f**) The positive rate of BrdU was compared. (**g**) Wound healing assays were performed at 0, 12, and 24 h. (**h**) Wound healing assay data represent the percentage of cell area at 12 and 24 h. (scale bar: 200 μm). Significant increases compared with the control: *p < 0.05, **p < 0.01. Significant decreases compared with MB addition and LED irradiation: ^†^*p* < 0.05, ^††^*p* < 0.01.

**Figure 6:**
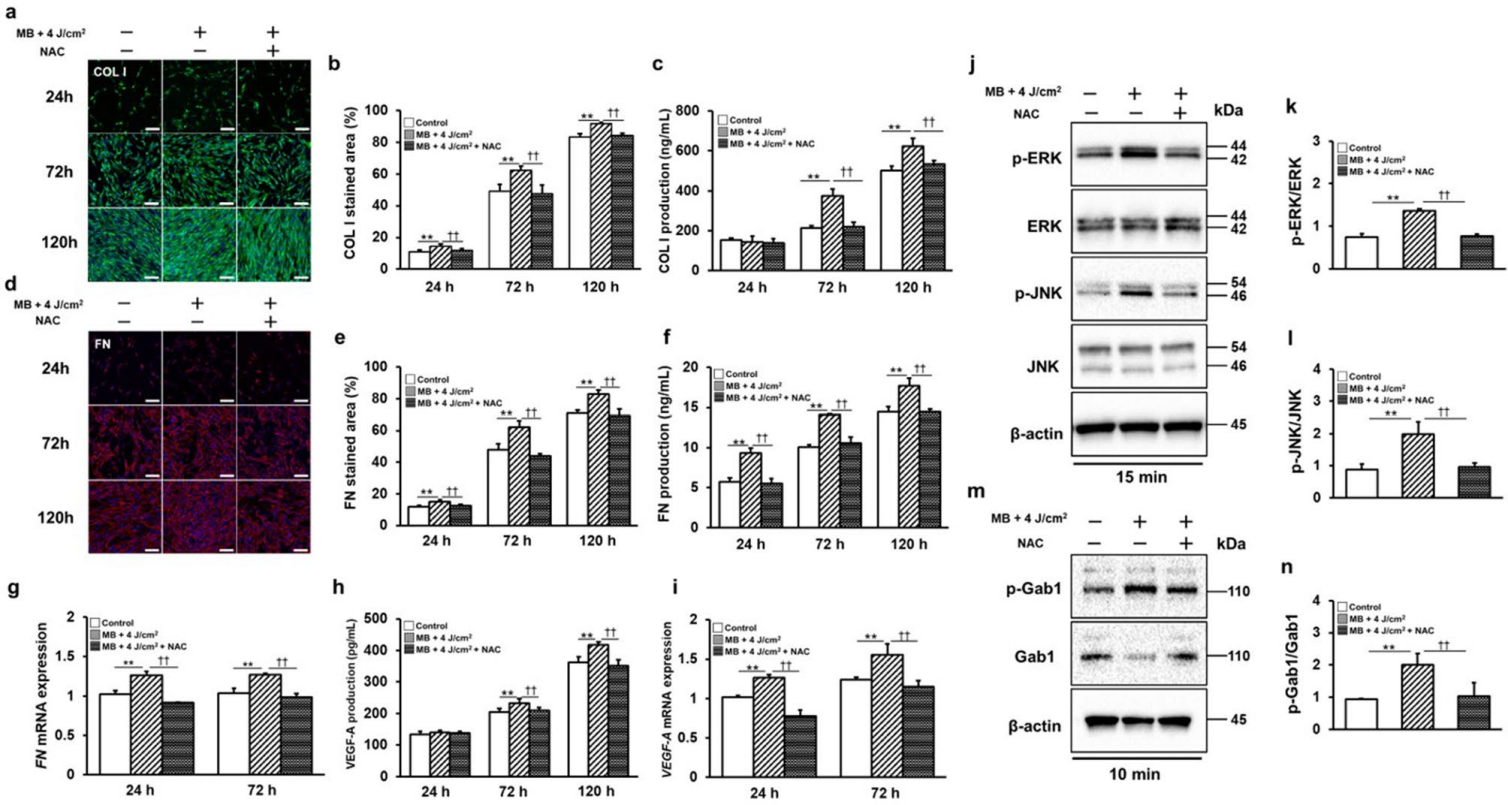
NAC inhibited the phosphorylation of the ROS-Gab1/ERK signaling pathway and suppressed the production of wound healing factors. (**a**, **b**) Immunofluorescence staining of COL I was visualized by confocal laser microscopy after 24, 72, and 120 h of incubation; COL I data were compared as a percentage of the stained area. (**c**) COL I production was measured at 24, 72, and 120 h. (**d**, **e**) Immunofluorescence staining of FN was visualized by confocal laser microscopy after 24, 72, and 120 h of incubation. The fluorescence intensity of FN was compared based on the ratio of staining area. (**f**) FN production was measured at 24, 72, and 120 h. (**g**) *FN* mRNA gene expression was measured at 24 and 72 h. (**h**) VEGF-A production was measured at 24, 72, and 120 h. (**i**) *VEGF-A* mRNA gene expression was measured at 24 and 72 h. The expression of the ERK1/2 and JNK signaling pathways was quantified by western blotting (**j**). p-ERK, ERK (**k**), p-JNK, and JNK (l) expression were quantified by ImageJ. Gab1 expression was quantified by western blotting (**m**) and by ImageJ (**n**). Western blot analysis was performed on protein extracts of these cells with antibodies against the indicated proteins with β-actin as a loading control. The samples derive from the same experiment and those gels/blots were processed in parallel. Uncropped blots for this experiment are presented in supplementary file 1. (scale bar: 200 μm). Significant increases compared with the control: *p < 0.05, **p < 0.01. Significant decreases compared with MB addition and LED irradiation: ^†^*p* < 0.05, ^††^*p* < 0.01.

To evaluate the effect of the ROS produced in the photosensitization reactions, N-Acetyl-L-cysteine (NAC), a ROS inhibitor, was used. We found that NAC treatment significantly decreased MB addition and LED irradiation-induced ROS levels, and that treatment with 10 mM NAC blocked the effect of PDT (Fig. 5a). NAC treatment significantly decreased MB addition-and LED irradiation-induced cell viability (Fig. 5b), calcein staining (Fig. 5c,d), cell proliferation (Fig. 5e,f), and cell migration (Fig. 5g,h). NAC treatment significantly reduced COL I (Fig. 6a,b,c) and FN (Fig. 6d,e,f) synthesis, and VEGF-A production (Fig. 6h) in PDT and suppressed *FN* (Fig. 6g) and *VEGF-A* (Fig. 6i) gene expression. Furthermore, ERK1/2 and JNK phosphorylation were significantly inhibited 15 min after stimulation (Fig. 6j,k,l). Gab1 expression was significantly inhibited after 10 min (Fig. 6m,n).

## Discussion

PDT involves the irradiation of a light source against photosensitizers. Although the responses to PDT vary depending on the target tissue, it can result in cell apoptosis by inducing a strong stress response^27^ or promoting healing by activating cells^28^. Jang et al.^29^ reported that the highest cell proliferative activity in human dermal fibroblasts was observed after 12 h of laser irradiation (3 J/cm^2^) with 5-aminolevulinic acid (ALA) (0.1 mM), a photosensitizer. Yang et al.^6^ reported that diode laser (44.3 J/cm^2^) irradiation of MB (5 µM) significantly enhanced HGnF migration after 24 h. In the present study, high-intensity red LED irradiation of HGnFs in the presence of MB promoted cell proliferation and migration most significantly at 4 J/cm^2^. The photodynamic reaction at 8 J/cm^2^ also showed an increasing trend for cell proliferation and migration, but a decreasing trend compared to that at 4 J/cm^2^. Although it does not cause cytotoxicity, which causes the LDH in the cytoplasm to flow out of the cell, it may exceed the parameters that have a beneficial effect on the cell. These results indicate that the photodynamic response induced by irradiation with photosensitizers with an adjusted light-source energy level promoted cell proliferation and migration. The effects of PDT on cells have been reported to vary depending on various factors, such as the composition and concentration of the photosensitizer, as well as the wavelength, density, dose, and output of the light source^14^. This suggests that it is important to determine the optimal irradiation parameters depending on the target tissue and the method of use. Although cytotoxic effects of MB, the photosensitizer in this study, have been reported at concentrations exceeding 100 µM^30^, no cytotoxicity was observed due to the low concentration of 3.126 µM. The light used in this study had a lower energy fluence rate than the laser. Although the difference between the effects of lasers and LED, which have different energy fluence rates, on cells, continues to be debated, the possibility of replacing lasers with LED without worsening the results has been suggested^7^. LED are compact, easy to handle, inexpensive, and safe for use. In addition, LED can irradiate a wider area than the current laser devices and are considered more effective for therapeutic applications.

Wound healing involves a series of inflammatory, proliferative, and remodeling phases in which the wound is replaced by granulation tissue to form new connective tissue. In gingival wound healing, fibroblasts migrate to the wound site and play a role in wound contraction and healing by synthesizing ECM^31^. Collagen is one of the most common proteins in the ECM, and COL I plays an important role in tissue healing by promoting tissue strength and scaffolding for cell adhesion and migration^32^. FN is also important for ECM formation and re-epithelialization. The polymerization of FN and collagen leads to changes in ECM composition, stability, and adhesion^33^. Yang et al.^6^ reported that diode laser irradiation with MB significantly increases COL I and FN expression. In the present study, COL I and FN levels were significantly increased. Vascular endothelial growth factor (VEGF)-A is an important factor that promotes angiogenesis during wound healing. The restoration of blood flow to the tissues is believed to provide the oxygen and nutrients needed to support the growth and function of the cells being repaired^34^. Yang et al.^35^ reported that irradiation with red LED (6 J/cm^2^) and ALA (0.1 mM) significantly increased VEGF-A production. In the present study, VEGF-A levels were significantly increased. These results suggest that photodynamic reactions promote ECM production and angiogenesis, leading to the formation of new granulation tissue, which in turn forms the basis for remodeling, thereby promoting gingival wound healing.

The MAPK signaling pathway regulates diverse cellular processes, such as cell proliferation, differentiation, and apoptosis, in response to stimuli such as growth factors and oxidative stress^36^. The MAPK pathway is comprised of a large family of proteins, including ERK1/2, JNK, and p38. The MAPK pathway mediates both mitogen-activated and stress-activated signals and is thus involved in intracellular redox regulation. Photodynamic reactions affect intracellular redox reactions, which may in turn affect the MAPK pathway. The ERK1/2 pathway is involved in several cellular responses including motility, differentiation, and survival^34^. The ERK1/2 pathway is also involved in cell migration and ECM production during wound healing and regulates cell metabolism, function, and tissue regeneration^3^. Jang et al.^29^ reported that the photodynamic reactions of ALA with diode laser irradiation resulted in prolonged activation of ERK1/2 and a significant increase in COL I. The JNK pathway has been reported to be involved in apoptosis as a stress activation signal as well as in cell proliferation, migration, and VEGF-A production in wound healing^37^. In the past, stimulation of growth factors was reported to activate the ERK1/2 and JNK pathways and promote wound healing^24,37^. The ERK1/2 and JNK pathways are important intracellular signaling pathways in the wound healing process. In the present study, both ERK1/2 and JNK signaling pathways were activated by a photodynamic reaction using high-intensity red LED irradiation. This suggests that the ERK1/2 and JNK pathways may be involved in the promotion of cell proliferation and migration, as well as in the production of wound-healing factors by photodynamic reactions at controlled energy doses.

Gab1 is recruited intracellularly to the plasma membrane by binding growth factors, extracellular ligands, and growth factors to RTKs, followed by phosphorylation^25,26^. It then acts as a scaffold, allowing for the transfer of phosphorylated RTKs to the cytoplasm^38^. It has been reported that stimulation with hepatocyte growth factor (HGF), a growth factor that activates Gab1, subsequently activates the ERK1/2 pathway^39^. Gab1 has been reported to be involved in intracellular signaling pathways by fibroblast growth factor (FGF) and epidermal growth factor (EGF)^40^. In this study, Gab1 was activated via photodynamic reactions by using a high-intensity red LED. This suggests that similar to the phosphorylation pathway triggered by ligand growth factors, photodynamic reactions trigger Gab1 binding and initiate intracellular signaling. Although RTKs present in the plasma membrane are always activated, the subsequent transmission of phosphorylation is always inhibited^41^. Stimulation by ligands such as growth factors and control inhibition allows phosphorylation to be transmitted to Gab1.

ROS plays a major role in this mechanism^23^. ROS is a general term for chemical species containing partially reduced oxygen, and are believed to induce cytotoxicity and genotoxicity, damaging lipids, proteins, and DNA. However, recent studies have shown that ROS function is regulated by the intracellular redox status. While excessive ROS production leads to apoptotic cell death, specific ROS levels are needed to regulate diverse cellular processes, such as cell proliferation^20^. ROS can alter the structure and function of proteins by modifying critical amino acid residues^42^. Oxidation of cysteine residues inactivates phosphatases and controls the inhibition of RTKs^43^. When growth factors act on cells, they generate intracellular ROS that allow for phosphorylation in the cytoplasm^23^. When ROS production is inhibited, the cellular response to growth factors is also inhibited. Several studies have reported that UV-induced production of ROS, independent of the ligand, mediates the activity of growth factor receptors in intracellular signaling^44,45^. It has also been reported that wound healing is accelerated by ROS generated by stimulation with H_2_O_2_^46^. In the present study, the inhibition of ROS production by photodynamic reactions using high-intensity red LED inhibited Gab1 and the MAPK pathway, resulting in the inhibition of cell proliferation and migration, as well as the production of wound healing factors. ROS produced by photodynamic reactions using LED irradiation with controlled energy levels have been suggested to phosphorylate Gab1 independent of the ligand, thereby enabling the activity of the MAPK pathway. Furthermore, by adjusting the irradiation energy level, the photodynamic reaction induced by high-intensity red LED promotes wound healing, suggesting that ROS play a significant role in this process. These results indicate that research should focus on exploring the positive effects of high-intensity red LED on the surrounding tissues to promote wound healing, rather than the no-effect method currently used in photodynamic therapy for periodontal disease treatment.

## Material and methods

### Cell culture

HGnFs were obtained from ScienCell Research Laboratories (San Diego, CA, USA) in Dulbecco’s modified eagle medium (DMEM) (Nacalai Tesque, Kyoto, Japan) supplemented with 10% fetal bovine serum (Thermo Fisher Scientific, Rockford, IL, USA), 500 U/mL penicillin, 500 U/mL streptomycin, and 0.25 µg/mL amphotericin B (Nacalai Tesque) at 37 °C in a 5% CO_2_ atmosphere.

### Irradiation procedure

An LED prototype emitter (LZ1-00R205 Deep Red LED; LedEngin, Santa Clara, CA, USA) was used, which emits red light specifically at wavelengths of 600–700 nm at a peak of 650 nm. An intensity of 1100 mW/cm^2^ was used. The distance from the LED to the cell layer was 22 mm, with a spot size of 4 cm^2^, and the total absolute irradiance was 400 mW/cm^2^. The total radiant exposures were 2, 4, 8, and 12 J/cm^2^ for 5, 10, 20, and 30 s, respectively, with continuous output. At the appropriate time point, the culture medium on the cells was replaced with the reagent, and irradiation was performed in the dark only once. The temperature changes in the reagent over these irradiation periods were compared with those in the controls (data not shown).

### Photosensitizer

MB (Nacalai Tesque) was used as a photosensitizer and was diluted in DMEM to 1 µg/mL (3.126 µM) for each experiment. The culture medium was replaced with a medium containing MB, allowed to stand for 10 min, and irradiated with an LED. After an additional 10 min of incubation, the cells were washed twice with PBS and incubated in a culture medium. The cells were then divided into four groups: untreated (control), MB medium only (MB), LED irradiation only (4 J/cm^2^), MB, and LED irradiation.

### Cell proliferation and cell migration

Live cells were stained with calcein acetoxymethyl ester (calcein-AM) and photographed using a BZ-II all-in-one fluorescence microscope (Keyence Corporation, Osaka, Japan). Cell viability assays were performed using Cell Titer Blue Reagent (Promega USA) and a microplate reader (Molecular Devices, Sunnyvale, CA, USA). The cell proliferation rate was measured by incorporating 5-bromo-2′-deoxyuridine (BrdU; Nacalai Tesque) into the DNA. Medium containing 10 μM BrdU was then added to each well and the plates were incubated for 2 h. After fixing and permeabilizing the cells, the DNA was hydrolyzed with 2 M HCL. Nuclear staining was performed with 4′,6-diamidino-2-phenylindole (DAPI) (Dojindo, Kumamoto, Japan) and then photographed with Keyence BZ-II all-in-one. The ImageJ software was used to calculate the percentage of BrdU-positive cells.

Cell migration assay was performed using a wound repair assay kit (Ibidi GmbH, Martinsried, Germany).

### Cell cytotoxicity assay

Lactate dehydrogenase (LDH) activity was measured in the control, MB, and MB + LED treatments. Triton (0.2%, Sigma-Aldrich, St. Louis, MO, USA) was used as a positive control. LDH activity was measured using the Cell Cytotoxicity LDH Assay Kit (Dojindo).

### Type I collagen, fibronectin and VEGF-A production

Following stimulation, the cells were incubated with primary antibodies against primary mouse anti-COL I antibody (sc-59772; (Santa Cruz Biotechnology, Texas, USA) and primary rabbit anti-FN antibody (ab 2413; Abcam, Cambridge UK) and incubated overnight at 4 °C. Nuclei were stained with DAPI, and fluorescence immunostaining was performed using Alexa Fluor 488® (Thermo Fisher Scientific, Waltham, MA, USA). COL I production in the supernatant was determined using the Procollagen Type I C-peptide (PIP) EIA Kit (Takara Bio, Shiga, Japan). FN production in the supernatant was determined using the FN Human ELISA Kit (Thermo Fisher Scientific). VEGF-A production in the supernatant was determined using the VEGF-A Human ELISA Kit (Thermo Fisher Scientific) according to the manufacturer’s protocol.

### Gene expression

Total RNA was isolated using an RNeasy Mini Kit (Qiagen, Venlo, Netherlands) and reverse-transcribed to complementary DNA using a Prime Script Reagent Kit (Takara Bio, Shiga, Japan). The expression of *VEGF-A* and *fibronectin* (*FN*) (TaqMan gene expression assay; Applied Biosystems, Thermo Fisher Scientific, VEGF-A; Hs00900055_m1 and FN 1; Hs01549976_m1) was quantified using the QuantStudio 3 Real-Time PCR System (Thermo Fisher Scientific). Using the gene expression results from the negative control group, the ΔΔCt method and normalized to GAPDH was used to calculate the relative gene expression for each group.

### Western blot analysis

Total protein was extracted using RIPA buffer (Thermo Fisher Scientific) supplemented with a protease inhibitor reagent (Thermo Fisher Scientific). A BCA Protein Assay Kit (Thermo Fisher Scientific) was used to determine the total protein concentration. The protein samples were separated, transferred, and blocked. Membranes (Bio-Rad, Hercules, CA, USA) were incubated with primary antibodies (Cell Signaling Technology, Danvers, MA, USA) against p-ERK, ERK, p-JNK, JNK, p-Gab1 (Thy627), Gab1, and β-actin. The membranes were washed and incubated with secondary antibodies (Cell Signaling Technology). The immunoreactive bands were visualized using a chemiluminescence kit (Nacalai Tesque). Western blot data were analyzed using the ChemiDoc MP System (Bio-Rad) and ImageJ (version 1.53e) (Wayne Rasband and contributors, National Institutes of Health, USA).

### ROS detection and involvement

ROS levels were measured after HGnF stimulation using a Total ROS Detection Kit (Dojindo), according to the manufacturer's instructions. To determine the optimal concentration of the ROS inhibitor NAC (ChemScene LLC, Monmouth Junction, NJ, USA), NAC was diluted to three concentrations (1, 5, and 10 mM) in MB medium at 4 J/cm^2^ irradiation. ROS levels were measured using a Total ROS Detection Kit (Dojindo).

### Statistical analysis

Statistical analyses were performed using IBM SPSS Statistics (version 17) (IBM, Chicago, IL, USA). All data are presented as mean ± standard deviation (SD). One-way analysis of variance (ANOVA) with Tukey’s test was used to determine statistical significance. *P* < 0.05 was considered statistically significant.

### Supplementary Information

Supplementary Figures.

## Data Availability

The datasets generated and/or analyzed in the current study are available from the corresponding author upon reasonable request.
