# Author Correction: Photodynamic reactions using high-intensity red LED promotes gingival wound healing by ROS induction

**DOI:** 10.1038/s41598-024-53902-7

**Published:** 2024-02-09

**Authors:** Emika Minagawa, Nobuhiro Yamauchi, Yoichiro Taguchi, Makoto Umeda

**Affiliations:** Department of Periodontology, Osaka Dental University, 8-1 Kuzuhahanazono-cho, Hirakata, Osaka Japan

Correction to: *Scientific Reports* 10.1038/s41598-023-43966-2, published online 10 October 2023

The original version of this Article contained errors, Reference 7 and 30 were interchanged. The correct Reference 7 and 30 are listed below:

7. Heiskanen, V., Hamblin, MR. Photobiomopdulation: Lasers vs Light Emitting Diodes?. Photochem Photobiol Sci. 17, 1003–1017 (2018).

30. Tardivo, J, P. et al. Methylene blue in photodynamic therapy: From basic mechanisms to clinical applications. Photodiagnosis Photodyn Ther. 2, 175–91 (2005).

Also, Reference 7 was incorrectly cited as Reference 30 in the sentence:

The light used in this study had a lower energy fluence rate than the laser. Although the difference between the effects of lasers and LED, which have different energy fluence rates, on cells, continues to be debated, the possibility of replacing lasers with LED without worsening the results has been suggested^30^.

it now reads:

The light used in this study had a lower energy fluence rate than the laser. Although the difference between the effects of lasers and LED, which have different energy fluence rates, on cells, continues to be debated, the possibility of replacing lasers with LED without worsening the results has been suggested^7^.

Also, Reference 13 was incorrectly cited as Reference 12 in the sentence:

“It is also utilized to kill periodontal pathogens in the treatment of periodontal disease^12^ and has garnered attention owing to the risk of bacterial resistance to antimicrobial agents and safety in patients with systemic disease.”

it now reads:

“It is also utilized to kill periodontal pathogens in the treatment of periodontal disease^13^ and has garnered attention owing to the risk of bacterial resistance to antimicrobial agents and safety in patients with systemic disease.”

As a result, the subsequent References have been renumbered.

The original Article has been corrected.

