# Supplementary material for: Photodynamic reactions using high-intensity red LED promotes gingival wound healing by ROS induction
Source: Sci Rep. 2023 Oct 10;13:17081. doi: 10.1038/s41598-023-43966-2 (PMC10564724; doi:10.1038/s41598-023-43966-2)
Supplement: Supplementary file 1 — Supplementary Figures. [file 41598_2023_43966_MOESM1_ESM.pdf]

Supplementary file

Title page

Article title Photodynamic reactions using high-intensity red LED promotes gingival wound healing by ROS induction

Supplementary item: 29 Figures

Author information

Emika Minagawa; Department of Periodontology, Osaka Dental University, Osaka Japan

E-mail: minagawa-e@cc.osaka-dent.ac.jp

Nobuhiro Yamauchi; Department of Periodontology, Osaka Dental University, Osaka Japan

E-mail: yamauchi@cc.osaka-dent.ac.jp

Yoichiro Taguchi; Department of Periodontology, Osaka Dental University, Osaka Japan

E-mail: taguchi@cc.osaka-dent.ac.jp

Makoto Umeda; Department of Periodontology, Osaka Dental University, Osaka Japan

E-mail: umeda-m@cc.osaka-dent.ac.jp

Corresponding Author: Yoichiro Taguchi

Department of Periodontology, Osaka Dental University

8-1, Kuzuhahanazono-cho, Hirakata, Osaka, Japan

TEL: +81-72-864-3084

FAX: +81-72-864-3184

E-mail: taguchi@cc.osaka-dent.ac.jp

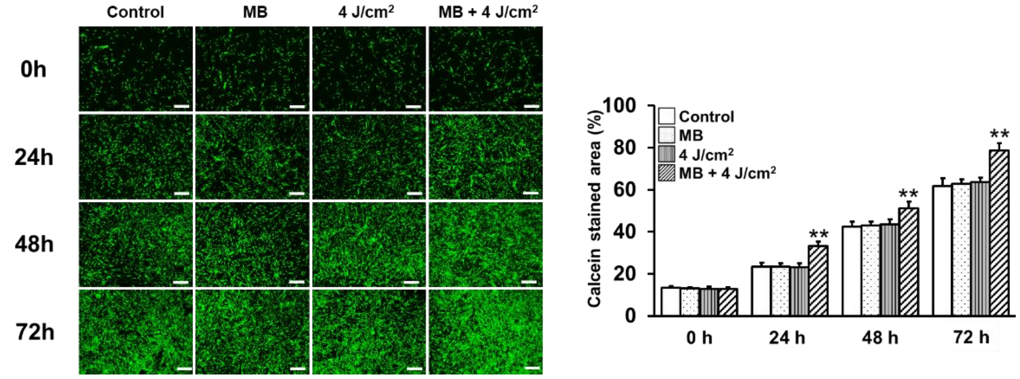

Supplementary Figure. 1

Calcein staining was visualized using fluorescence microscopy after 0, 24, 48, and 72 h of incubation and the percentages of calcein-stained areas were compared. 4 J/cm^2 alone showed no significant difference compared with the control. (scale bar: 200 μm)

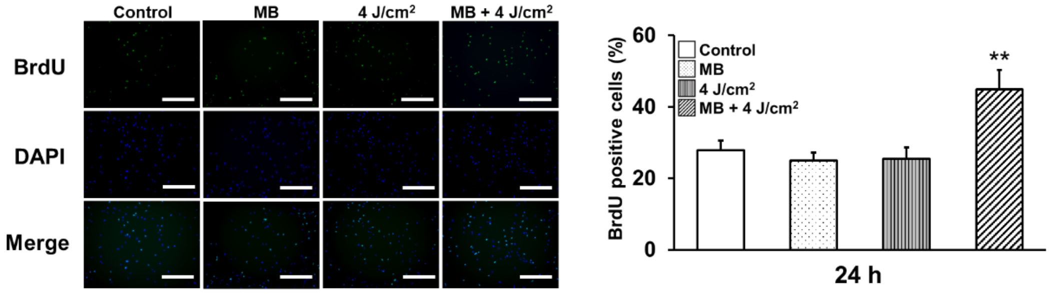

Supplementary Figure. 2

Cell proliferation was assessed by fluorescence immunostaining after 24 h of incubation and the rates of BrdU positivity are compared. 4 J/cm^2 alone showed no significant difference compared with the control. (scale bar: 200 μm)

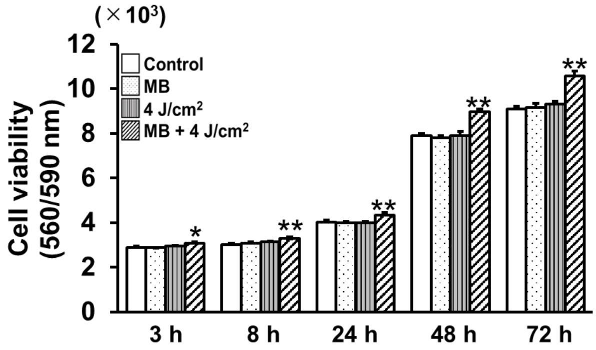

Supplementary Figure. 3

Cell survival was measured after 3, 8, 24, 48, and 72 h of incubation. 4 J/cm^2 alone showed no significant difference compared with the control.

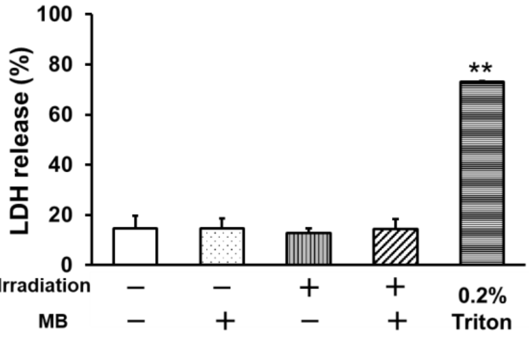

Supplementary Figure. 4

LDH production was measured after 72 h. 4 J/cm^2 alone showed no significant difference compared with the control.

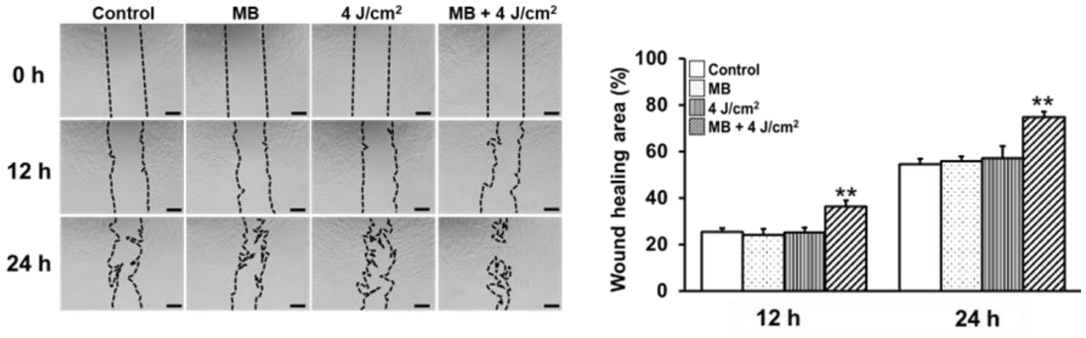

Supplementary Figure. 5

Wound healing assays were performed at 0, 12, and 24 h and wound healing assay data represent the percentage of cellular area at 12 and 24 h. 4 J/cm^2 alone showed no significant difference compared with the control. (scale bar: 200 μm)

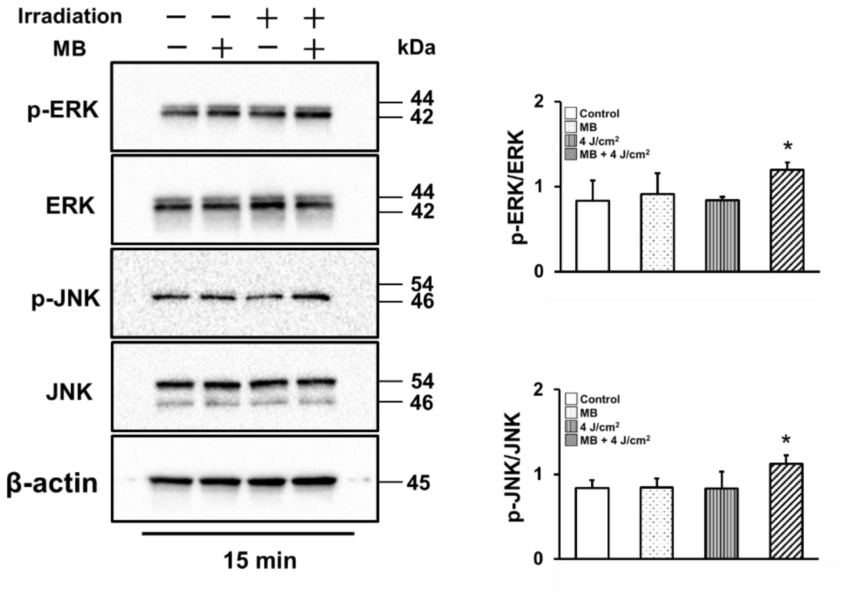

Supplementary Figure. 6

The expression levels of ERK1/2 and JNK were analyzed using western blotting. p-ERK, ERK, p-JNK, and JNK expressions were quantified using ImageJ software. 4 J/cm^2 alone showed no significant difference compared with the control.

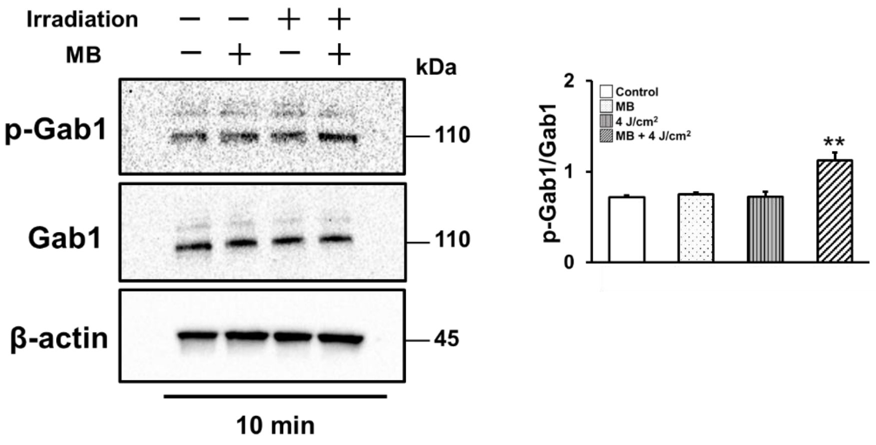

Supplementary Figure. 7

The expression levels of Gab1 was analyzed using western blotting. p-Gab1 and Gab1 expressions were quantified using ImageJ software. 4 J/cm^2 alone showed no significant difference compared with the control.

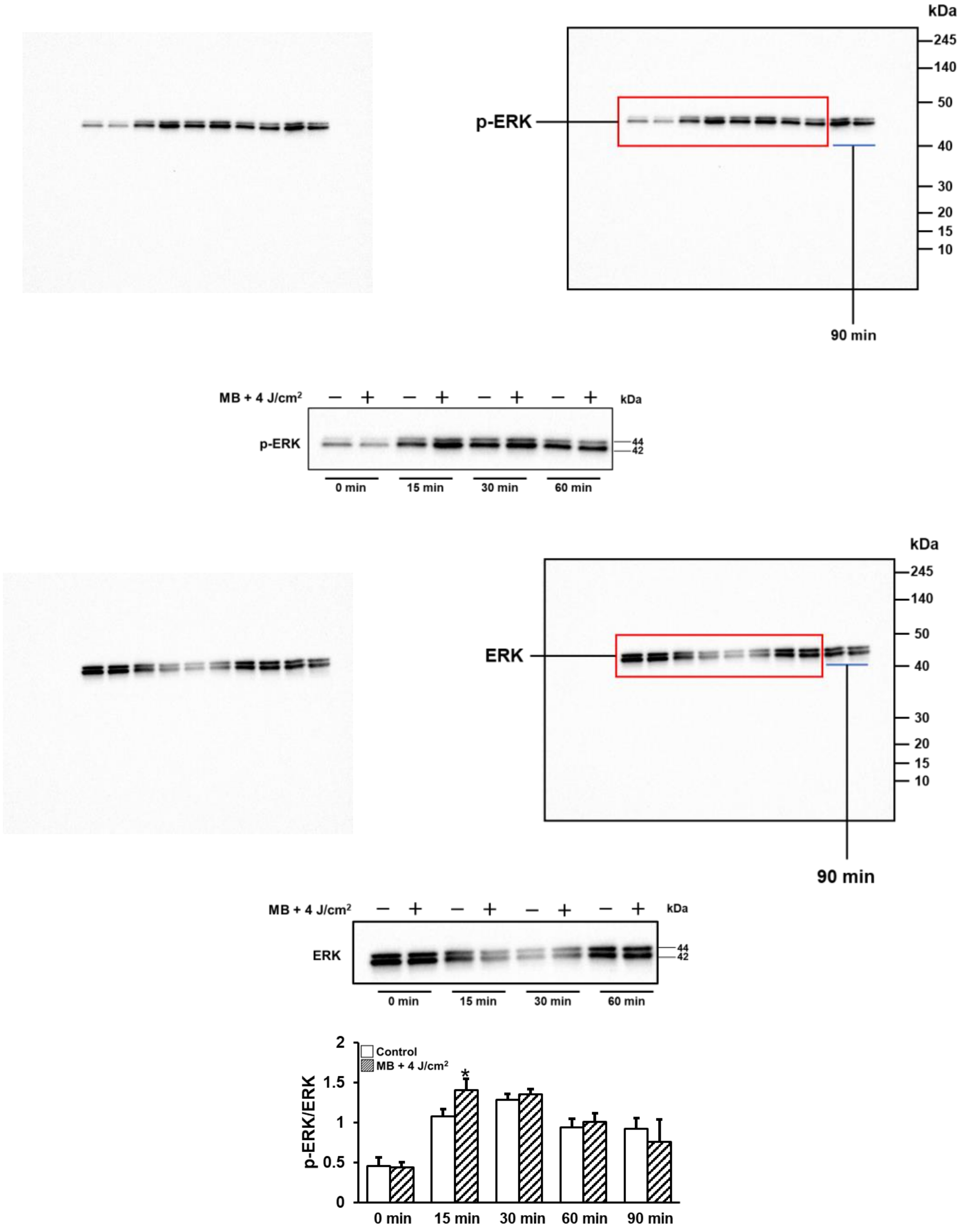

Supplementary Figure. 8

The original whole western blot of p-ERK (Figure 3a, on the panel below) exposed for 90s and ERK (Figure 3a, on the panel below) exposed for 90s. Since there was no significant difference 60 and 90 minutes compared to the control, the submission was made at 0, 15, 30, 60 minutes. For the final figure, the regions of the original blots have been denoted using red boxes. PowerPoint was used to crop the excess outside the red boxes.

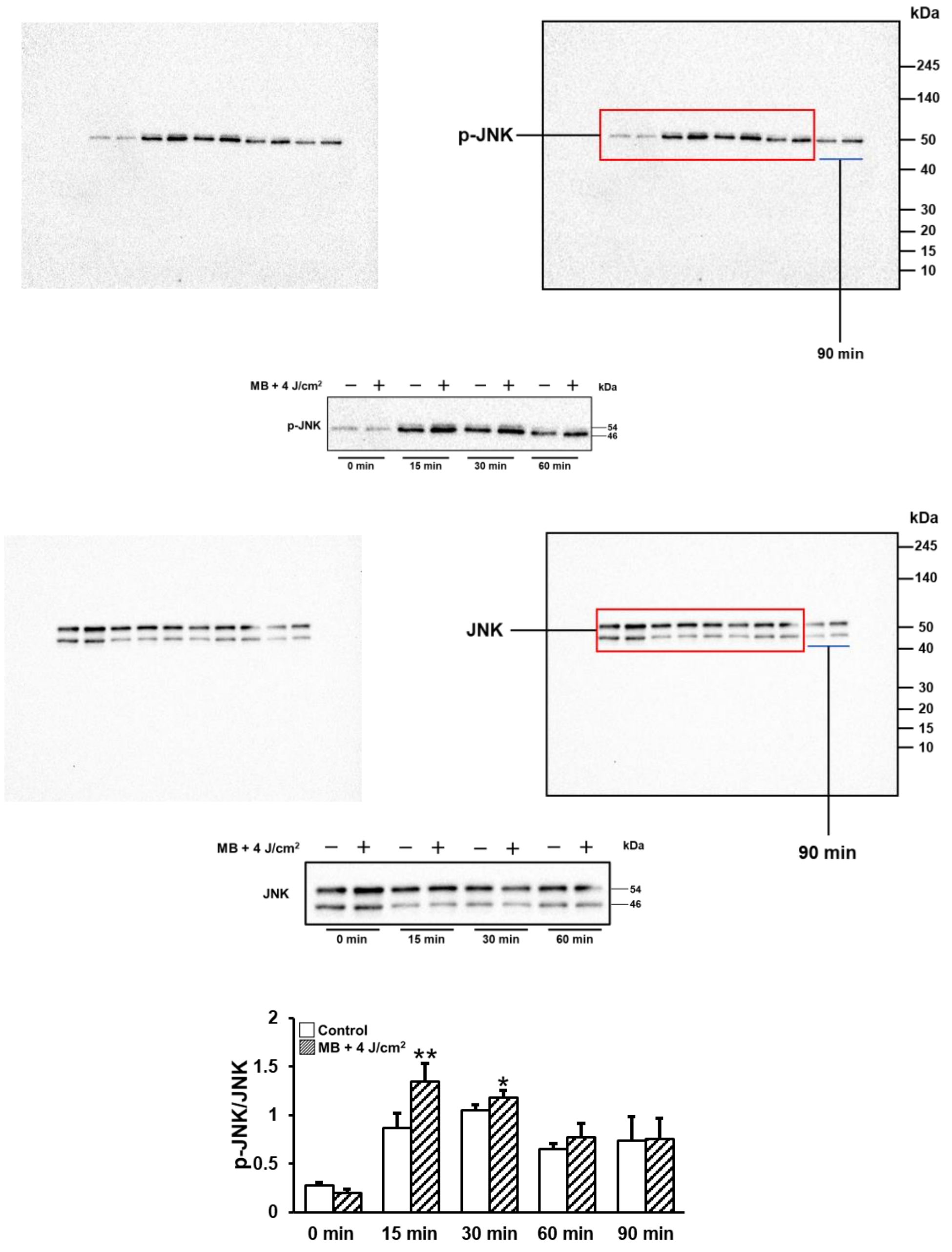

Supplementary Figure. 9

The original whole western blot of p-JNK (Figure 3a, on the panel below) exposed for 180s and JNK (Figure 3a, on the panel below) exposed for 300s. Since there was no significant difference 60 and 90 minutes compared to the control, the submission was made at 0, 15, 30, 60 minutes. For the final figure, the regions of the original blots have been denoted using red boxes. PowerPoint was used to crop the excess outside the red boxes.

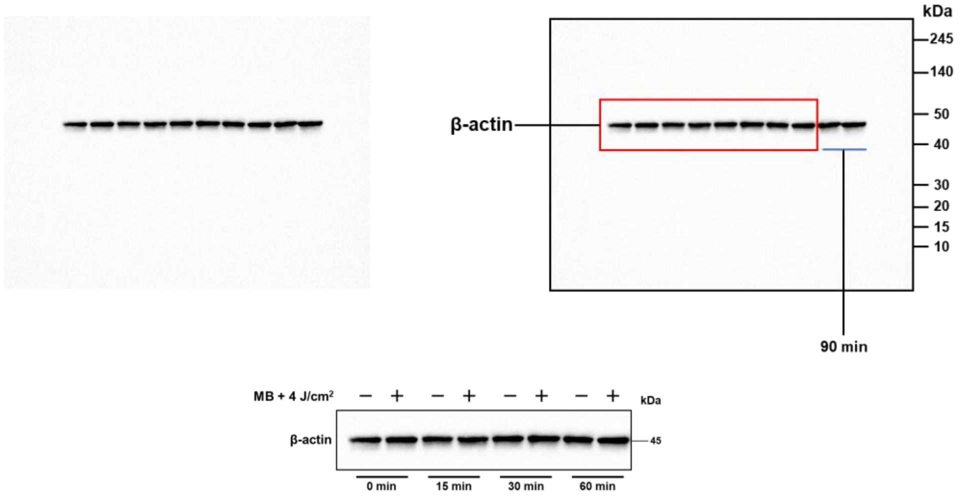

Supplementary Figure. 10

The original whole western blot of β-actin (Figure 3a, on the panel below) exposed for 10s. Since there was no significant difference 60 and 90 minutes compared to the control, the submission was made at 0, 15, 30, 60 minutes. For the final figure, the regions of the original blots have been denoted using red boxes. PowerPoint was used to crop the excess outside the red boxes.

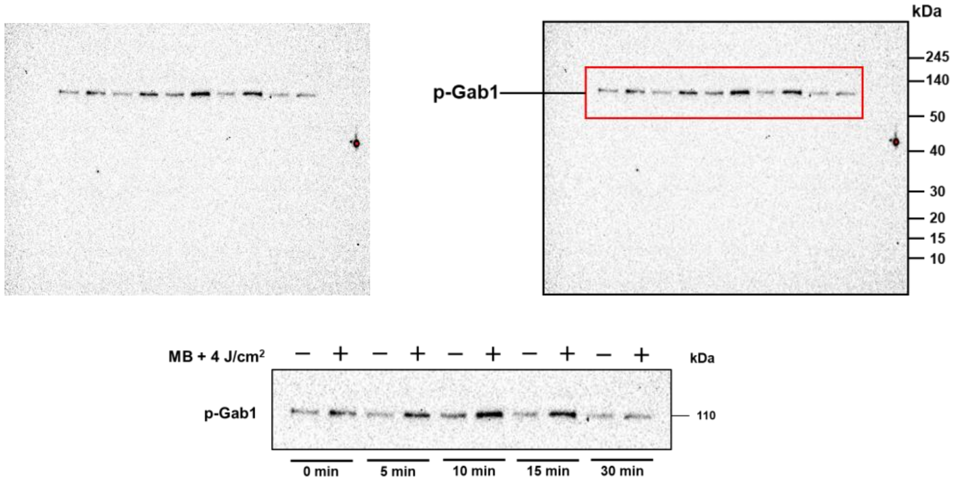

Supplementary Figure. 11

The original whole western blot of p-Gab1 (Figure 3d, on the panel below) exposed for 1200s. For the final figure, the regions of the original blots have been denoted using red boxes. PowerPoint was used to crop the excess outside the red boxes.

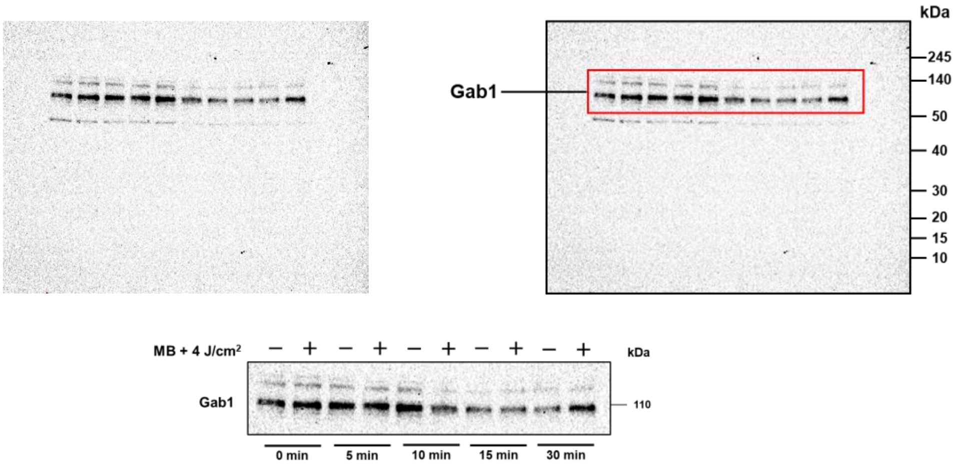

Supplementary Figure. 12

The original whole western blot of Gab1 (Figure 3d, on the panel below) exposed for 1200s. For the final figure, the regions of the original blots have been denoted using red boxes. PowerPoint was used to crop the excess outside the red boxes.

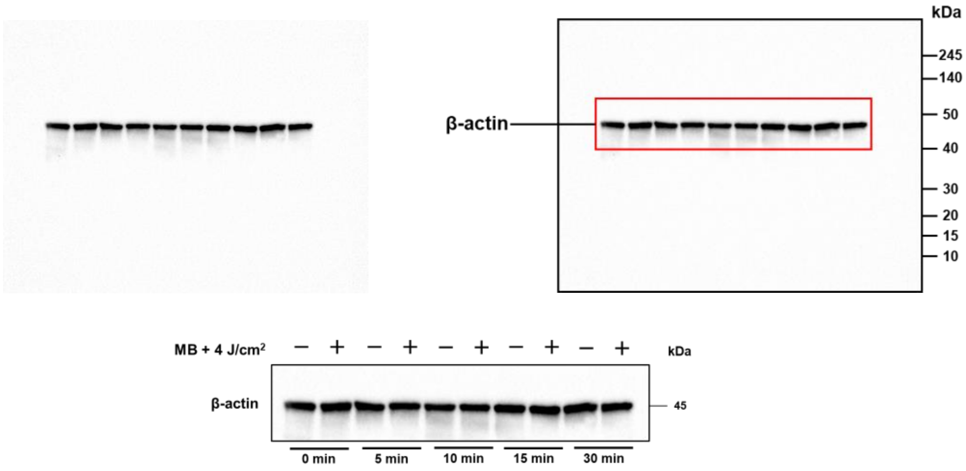

Supplementary Figure. 13

The original whole western blot of β-actin (Figure 3d, on the panel below) exposed for 10s. For the final figure, the regions of the original blots have been denoted using red boxes. PowerPoint was used to crop the excess outside the red boxes.

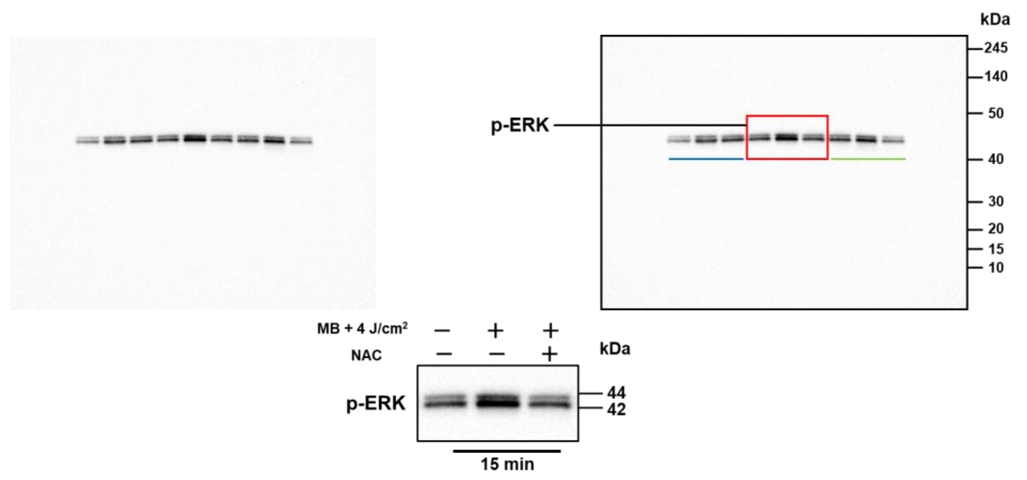

Supplementary Figure. 14

The original whole western blot of ERK (Figure 6j, on the panel below) exposed for 60s. It was done three times on one membrane to obtain significant differences. For the final figure, the regions of the original blots have been denoted using red boxes. PowerPoint was used to crop the excess outside the red boxes.

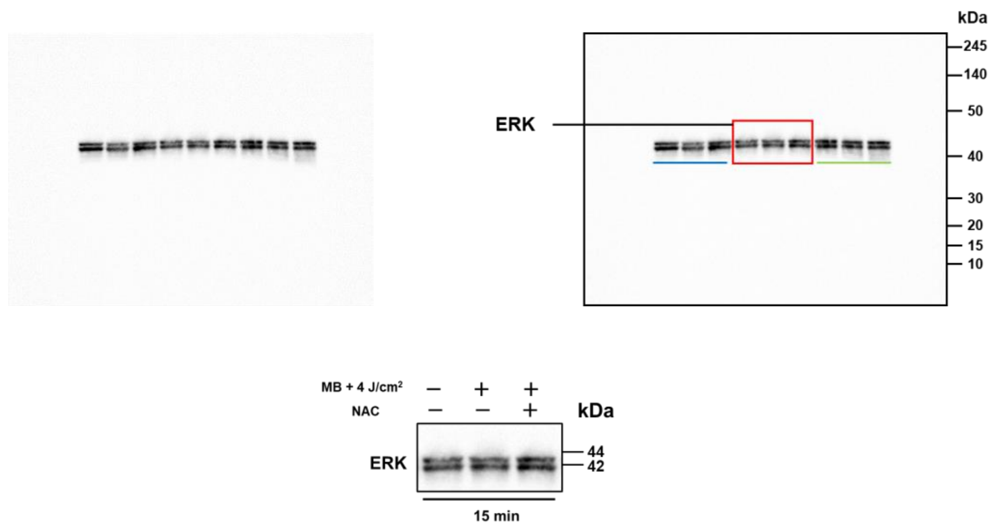

Supplementary Figure. 15

The original whole western blot of ERK (Figure 6j, on the panel below) exposed for 90s. It was done three times on one membrane to obtain significant differences. For the final figure, the regions of the original blots have been denoted using red boxes. PowerPoint was used to crop the excess outside the red boxes.

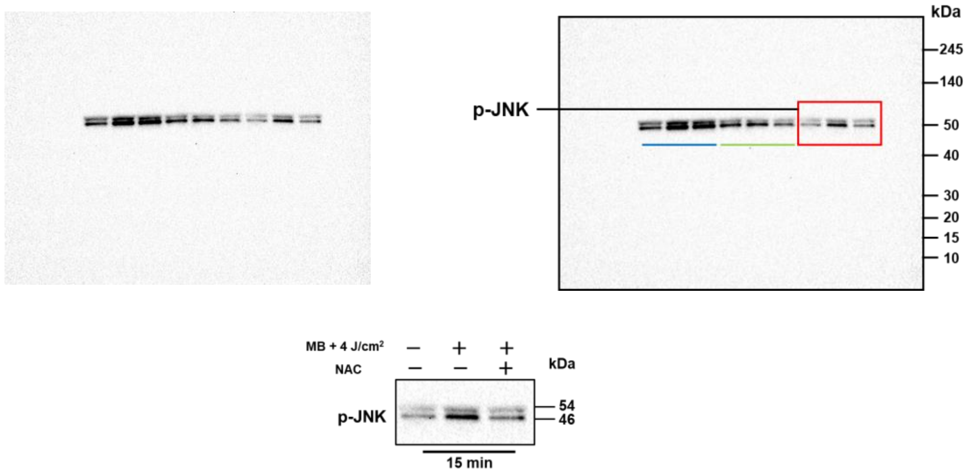

Supplementary Figure. 16

The original whole western blot of ERK (Figure 6j, on the panel below) exposed for 180s. It was done three times on one membrane to obtain significant differences. For the final figure, the regions of the original blots have been denoted using red boxes. PowerPoint was used to crop the excess outside the red boxes.

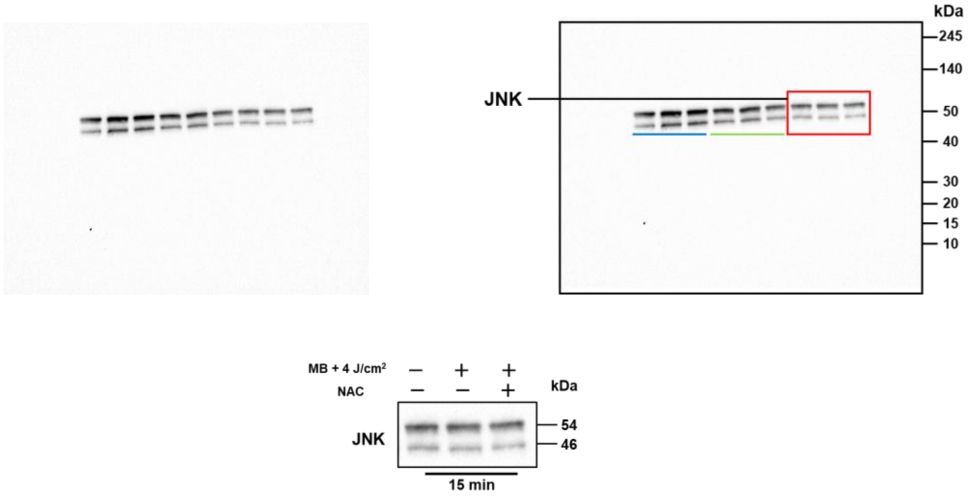

Supplementary Figure. 17

The original whole western blot of JNK (Figure 6j, on the panel below) exposed for 90s. It was done three times on one membrane to obtain significant differences. For the final figure, the regions of the original blots have been denoted using red boxes. PowerPoint was used to crop the excess outside the red boxes.

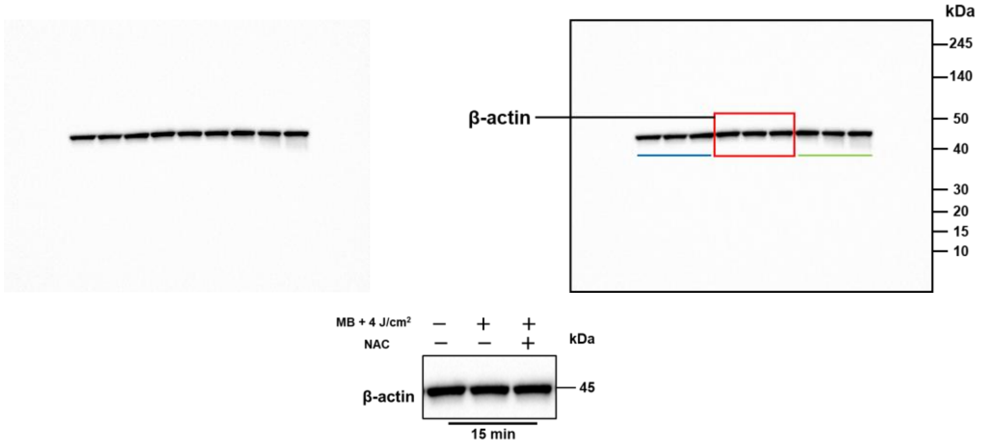

Supplementary Figure. 18

The original whole western blot of β-actin (Figure 6j, on the panel below) exposed for 10s. It was done three times on one membrane to obtain significant differences. For the final figure, the regions of the original blots have been denoted using red boxes. PowerPoint was used to crop the excess outside the red boxes.

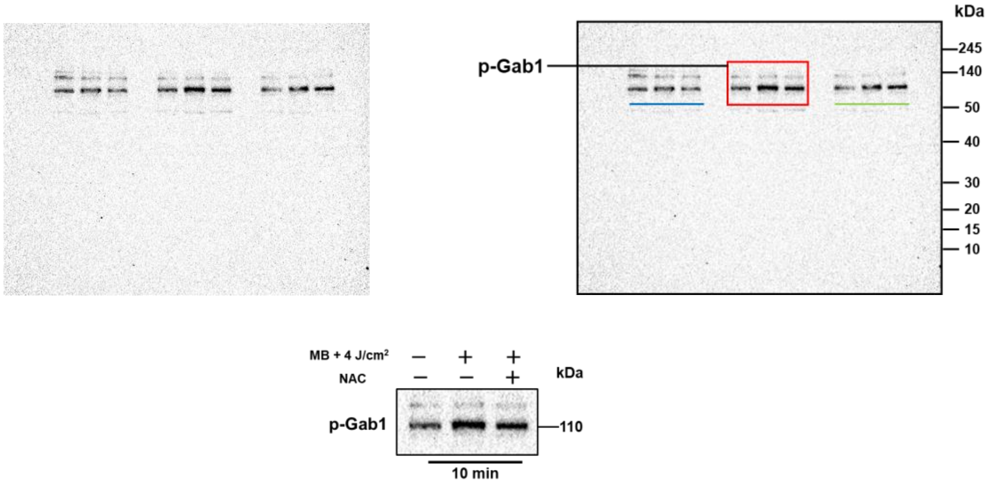

Supplementary Figure. 19

The original whole western blot of p-Gab1 (Figure 6m, on the panel below) exposed for 1200s. It was done three times on one membrane to obtain significant differences. For the final figure, the regions of the original blots have been denoted using red boxes. PowerPoint was used to crop the excess outside the red boxes.

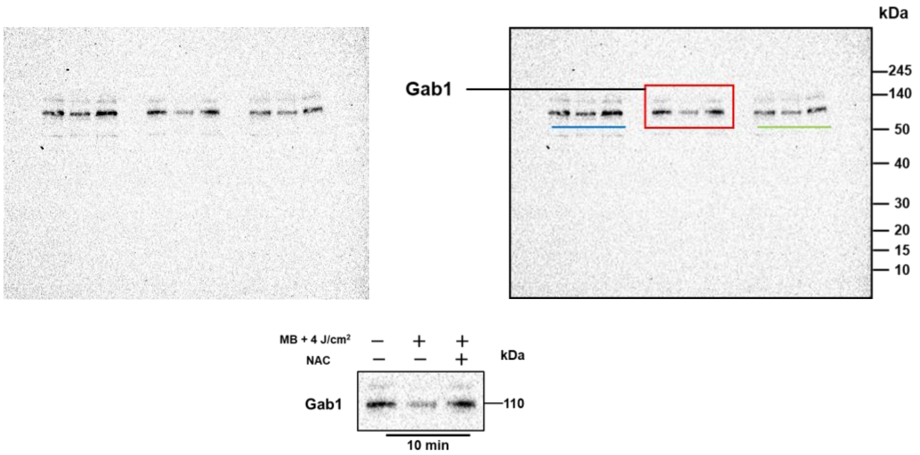

Supplementary Figure. 20

The original whole western blot of Gab1 (Figure 6m, on the panel below) exposed for 1200s. It was done three times on one membrane to obtain significant differences. For the final figure, the regions of the original blots have been denoted using red boxes. PowerPoint was used to crop the excess outside the red boxes.

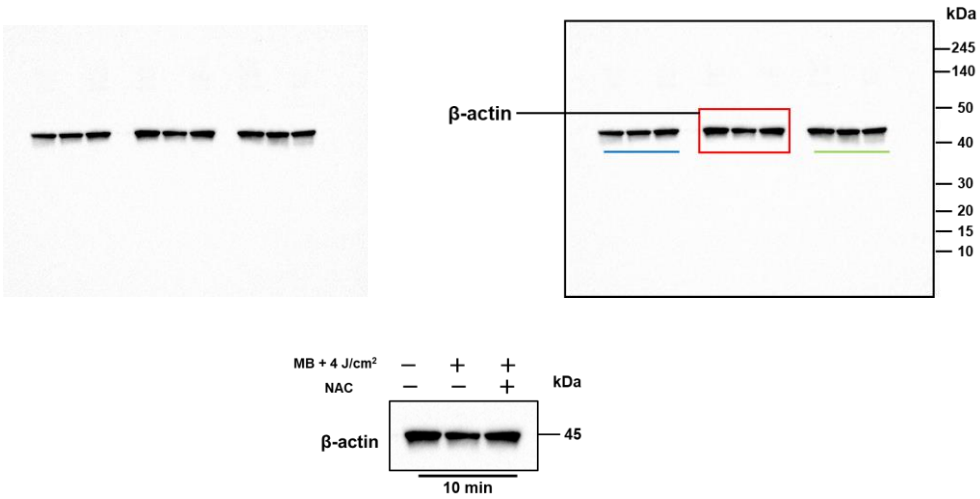

Supplementary Figure. 21

The original whole western blot of β-actin (Figure 6m, on the panel below) exposed for 10s. It was done three times on one membrane to obtain significant differences. For the final figure, the regions of the original blots have been denoted using red boxes. PowerPoint was used to crop the excess outside the red boxes.

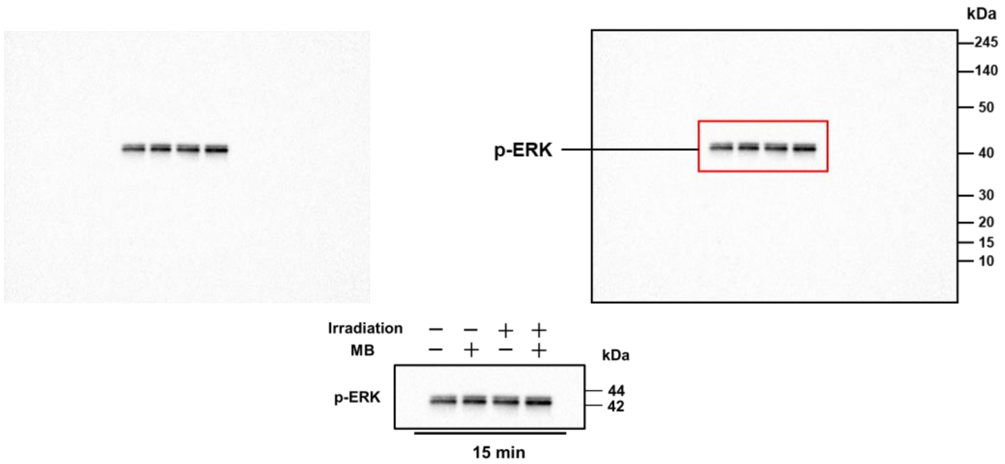

Supplementary Figure. 22

The original whole western blot of p-ERK (Supplementary Figure 6, on the panel below) exposed for 30s. For the final figure, the regions of the original blots have been denoted using red boxes. PowerPoint was used to crop the excess outside the red boxes.

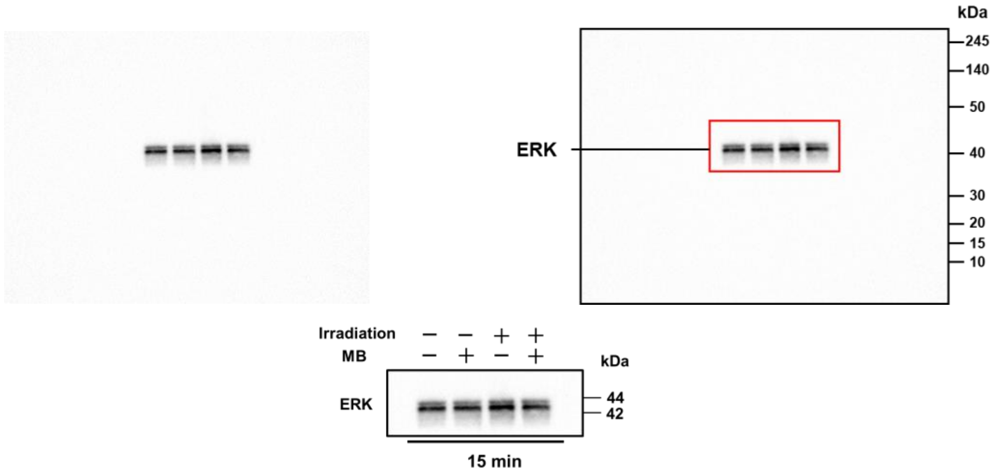

Supplementary Figure. 23

The original whole western blot of ERK (Supplementary Figure 6, on the panel below) exposed for 60s. For the final figure, the regions of the original blots have been denoted using red boxes. PowerPoint was used to crop the excess outside the red boxes.

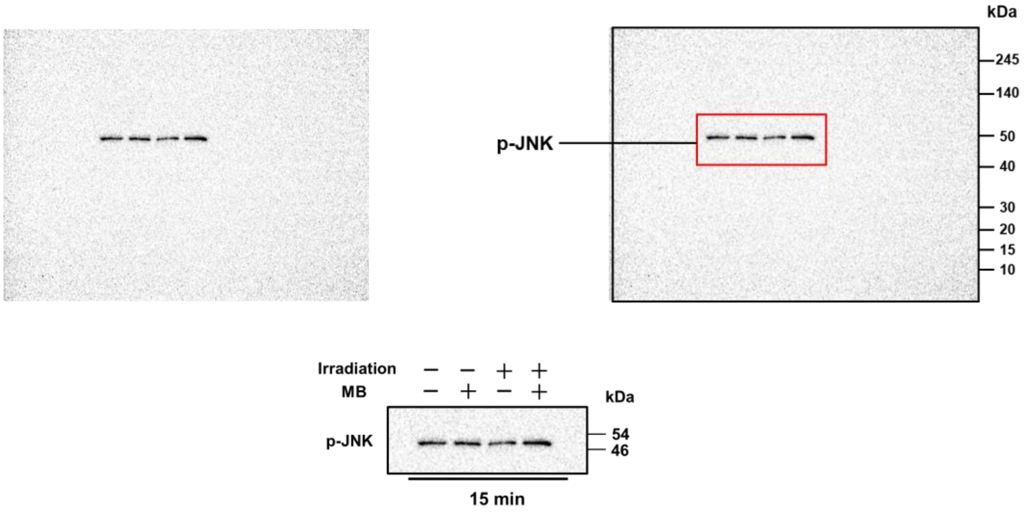

Supplementary Figure. 24

The original whole western blot of p-JNK (Supplementary Figure 6, on the panel below) exposed for 300s. For the final figure, the regions of the original blots have been denoted using red boxes. PowerPoint was used to crop the excess outside the red boxes.

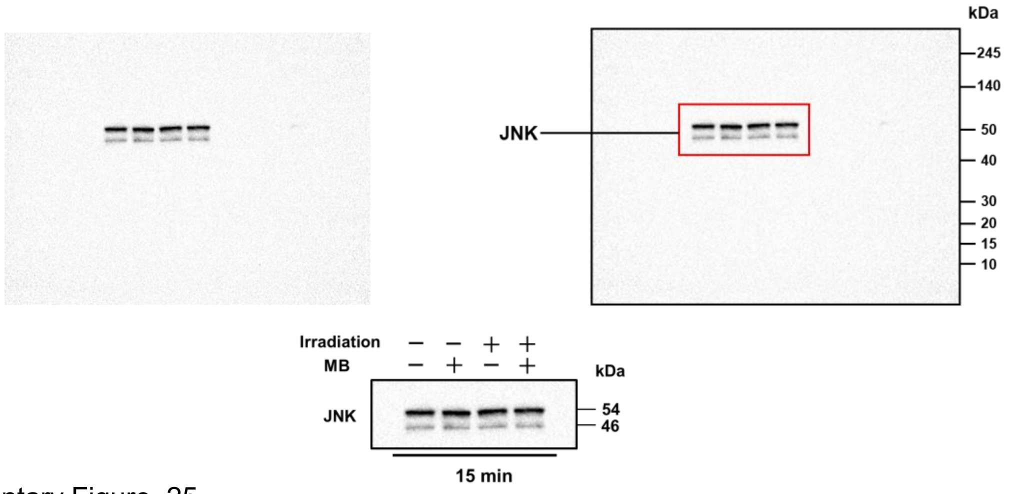

Supplementary Figure. 25

The original whole western blot of JNK (Supplementary Figure 6, on the panel below) exposed for 60s. For the final figure, the regions of the original blots have been denoted using red boxes. PowerPoint was used to crop the excess outside the red boxes.

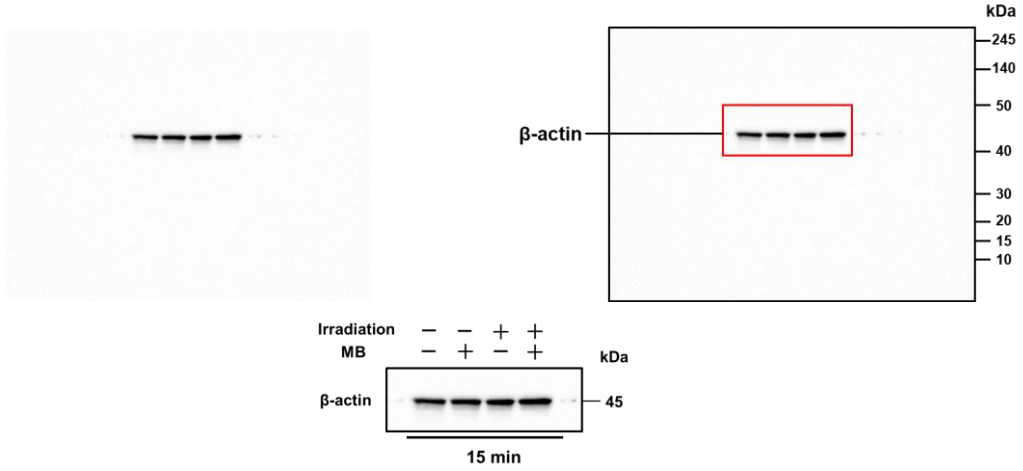

Supplementary Figure. 26

The original whole western blot of β-actin (Supplementary Figure 6, on the panel below) exposed for 10s. For the final figure, the regions of the original blots have been denoted using red boxes. PowerPoint was used to crop the excess outside the red boxes.

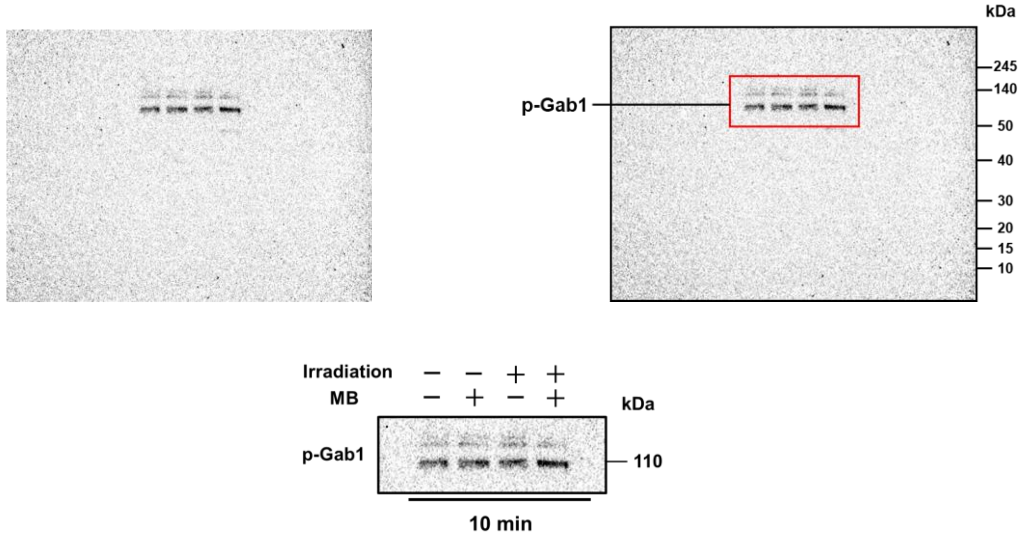

Supplementary Figure. 27

The original whole western blot of p-Gab1 (Supplementary Figure 7, on the panel below) exposed for 1200s. For the final figure, the regions of the original blots have been denoted using red boxes. PowerPoint was used to crop the excess outside the red boxes.

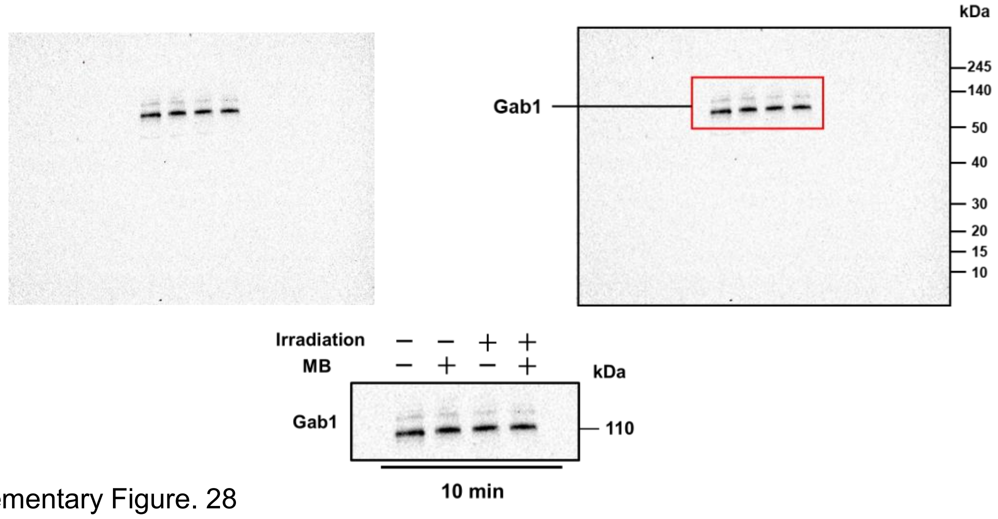

Supplementary Figure. 28

The original whole western blot of Gab1 (Supplementary Figure 7, on the panel below) exposed for 1200s. For the final figure, the regions of the original blots have been denoted using red boxes. PowerPoint was used to crop the excess outside the red boxes.

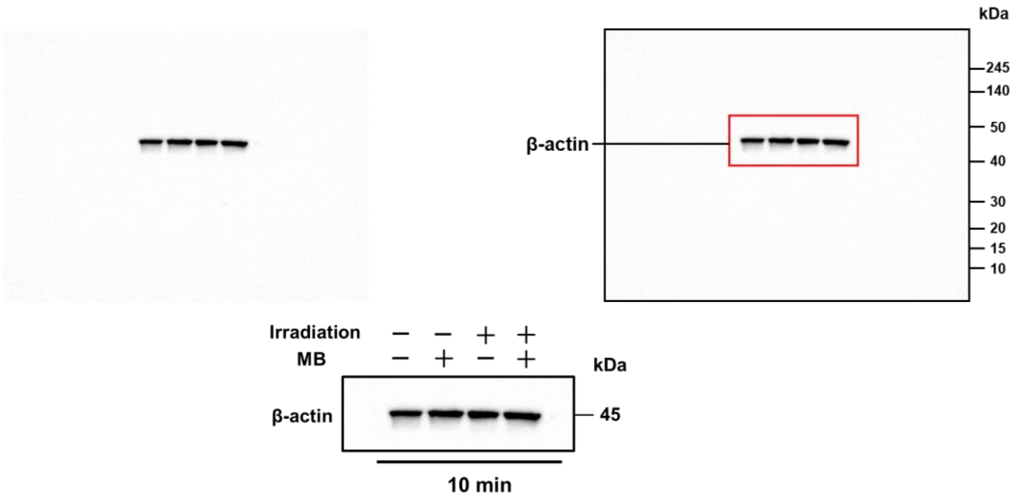

Supplementary Figure. 29

The original whole western blot of β-actin (Supplementary Figure 7, on the panel below) exposed for 10s. For the final figure, the regions of the original blots have been denoted using red boxes. PowerPoint was used to crop the excess outside the red boxes.
